# Supplementary material for: Genetic Diversity and Relationships Among Tunisian Wild and Cultivated Rosa L. Species
Source: Plants (Basel). 2024 Dec 20;13(24):3563. doi: 10.3390/plants13243563 (PMC11678506; doi:10.3390/plants13243563)
Supplement: Supplementary file 1 [file plants-13-03563-s001.zip › Table S1.pdf]

Table S1. List of sample names, code, Rose group and geographical origin of the studied rose accessions.

Figure3 code	Figure5 code	Sample name	Code	Group	Geographical origin
1	1	Rose of Ariana	RA.LK(1)	Perfume Rose	Ariana
2	2	Rose of Ariana	RA.LK(2)	Perfume Rose	Ariana
3	3	Rose of Ariana	RA.LK(3)	Perfume Rose	Ariana
4	4	Rose of Ariana	RA.LK(4)	Perfume Rose	Ariana
5	5	Rose of Ariana	RA.BNG(1)	Perfume Rose	NGBT
6	6	Rose of Ariana	RA.BNG(2)	Perfume Rose	NGBT
7	7	Rose of Ariana	RA.BNG(3)	Perfume Rose	NGBT
8	8	Rose of Ariana	RA.BNG(4)	Perfume Rose	NGBT
9	9	Rose of Ariana	RA.IB(1)	Perfume Rose	Ariana
10	10	Rose of Ariana	RA.IB(2)	Perfume Rose	Ariana
11	12	*Rosa centifolia*	RCe.IB(1)	Perfume Rose	Ariana
12	13	*Rosa centifolia*	RCe.IB(2)	Perfume Rose	Ariana
13	14	*Rosa centifolia*	RCe.IB(3)	Perfume Rose	Ariana
14	19	*Rosa damascena*	RD.SA(1)	Perfume Rose	GDA Sidi Amor
15	20	*Rosa damascena*	RD.SA(2)	Perfume Rose	GDA Sidi Amor
16	21	*Rosa damascena*	RD.SA(3)	Perfume Rose	GDA Sidi Amor
17	*26*	Rose of Kairouan	RK.Kh(1)	Perfume Rose	Kairouan (Khazzazia)
18	*27*	Rose of Kairouan	RK.Kh(2)	Perfume Rose	Kairouan (Khazzazia)
19	*28*	Rose of Kairouan	RK.Kh(3)	Perfume Rose	Kairouan (Khazzazia)
20	*29*	Rose of Kairouan	RK.DT(1)	Perfume Rose	Kairouan (Dhraa Tammar)
21	*30*	Rose of Kairouan	RK.DT(2)	Perfume Rose	Kairouan (Dhraa Tammar)
22	*31*	Rose of Kairouan	RK.DT(3)	Perfume Rose	Kairouan (Dhraa Tammar)
23	32	Rose of Kairouan	RK.R(1)	Perfume Rose	Kairouan (Raggeda)
24	*33*	Rose of Kairouan	RK.R(2)	Perfume Rose	Kairouan (Raggeda)
25	*34*	Rose of Kairouan	RK.R(3)	Perfume Rose	Kairouan (Raggeda)
26	*22*	*Rosa damascena*	RD.S(1)	Perfume Rose	Sfax (Aouabed)
27	*23*	*Rosa damascena*	RD.S(2)	Perfume Rose	Sfax (Aouabed)
28	*24*	*Rosa damascena*	RD.S(3)	Perfume Rose	Sfax (Aouabed)
29	*25*	*Rosa damascena*	RD.S(4)	Perfume Rose	Sfax (Sfax city)
30	35	Rose of Kairouan	RK.IB(1)	Perfume Rose	Ariana
31	36	Rose of Kairouan	RK.IB(2)	Perfume Rose	Ariana
32	37	Rose of Kairouan	RK.IB(3)	Perfume Rose	Ariana
33	15	*Rosa centifolia*	RCe.SA(1)	Perfume Rose	GDA Sidi Amor
34	16	*Rosa centifolia*	RCe.SA(2)	Perfume Rose	GDA Sidi Amor
35	17	*Rosa centifolia*	RCe.SA(3)	Perfume Rose	GDA Sidi Amor
36	18	*Rosa centifolia*	RCe.SA(4)	Perfume Rose	GDA Sidi Amor
37	38	*Rosa gallica* - Rose de Provins	RG.RdP.SA	Perfume Rose	GDA Sidi Amor
38	39	Almadinah rose	RMé.SA	Perfume Rose	GDA Sidi Amor
39	11	Rose of Ariana	RA.SA	Perfume Rose	GDA Sidi Amor

Table S1. List of sample names, code, Rose group and geographical origin of the studied rose accessions (Continuation)

Figure 3 code	Figure 5 code	Sample name	Code	Group	Geographical origin
40	40	Old Rose	OR.LK	Perfume Rose	Ariana
41	41	Rose de Rescht	RdR.SA	Perfume Rose	GDA Sidi Amor
42	_	Lady Emma Hamelton	LEH.SA	English Modern Rose	GDA Sidi Amor
43	_	Eustacia Vye	EV.SA	English Modern Rose	GDA Sidi Amor
44	_	Benjamin-Britten	BB.SA	English Modern Rose	GDA Sidi Amor
45	_	Golden Celebration	GC.SA	English Modern Rose	GDA Sidi Amor
46	_	Gertrude Jekyll	GJ.SA	English Modern Rose	GDA Sidi Amor
47	_	Lady of Shalott	LOS.SA	English Modern Rose	GDA Sidi Amor
48	_	L.D.Braithwaite	LDB.SA	English Modern Rose	GDA Sidi Amor
49	_	Graham Thomas	GT.SA	English Modern Rose	GDA Sidi Amor
50	_	Brother Cadfeal	BC.SA	English Modern Rose	GDA Sidi Amor
51	_	The Alnwick rose	TAR.SA	English Modern Rose	GDA Sidi Amor
52	_	Grace	G.SA	English Modern Rose	GDA Sidi Amor
53	_	Princess Alexandra of Kent	PAK.SA	English Modern Rose	GDA Sidi Amor
54	_	The mayflower	TM.SA	English Modern Rose	GDA Sidi Amor
55	_	Sharifa Asma	ShA.SA	English Modern Rose	GDA Sidi Amor
56	_	Susan William Ellis	SWE.SA	English Modern Rose	GDA Sidi Amor
57	_	Leander	Le.SA	English Modern Rose	GDA Sidi Amor
58	_	Teasing Georgia	TG.SA	English Modern Rose	GDA Sidi Amor
59	_	The Pilgrim	TP.SA	English Modern Rose	GDA Sidi Amor
60	_	Hyde Hall	HH.SA	English Modern Rose	GDA Sidi Amor
61	_	Charlotte	Ch.SA	English Modern Rose	GDA Sidi Amor
62	_	Molineux	M.SA	English Modern Rose	GDA Sidi Amor
63	_	Mary Rose	MR.SA	English Modern Rose	GDA Sidi Amor
64	_	Bathsheba	B.SA	English Modern Rose	GDA Sidi Amor
65	_	Irene Watts	RCh.IW.SA	China	GDA Sidi Amor
66	_	Rosa chinensis Mutabilis	RChM.SA	China	GDA Sidi Amor
67	_	Philadelphus	Ph.SA	Climber	GDA Sidi Amor
68	_	Alexandre Girault	AG.SA	Hybrid Wichurana	GDA Sidi Amor
69	_	Paul Noel	PN.SA	Hybrid Wichurana	GDA Sidi Amor
70	_	Paul Transon	PT.SA	Hybrid Wichurana	GDA Sidi Amor
71	_	Guirland Rose	GR.SA	Hybrid Wichurana	GDA Sidi Amor
72	_	The Fairy	TF.SA	Polyantha	GDA Sidi Amor
73	_	Prestige de Belgrarde	PdB.SA	Polyantha	GDA Sidi Amor
74	42	*Rosa sempervirens*	RS.SA	Wild	GDA Sidi Amor
75	43	Buff Beauty	HM.BB.SA	Moschata	GDA Sidi Amor
76	44	Cléance et Rosalie	HM.CR.SA	Moschata	GDA Sidi Amor
77	45	Ballerina	HM.B.SA	Moschata	GDA Sidi Amor

Table S1. List of sample names, code, Rose group and geographical origin of the studied rose accessions (Continuation)

Figure 3 code	Figure 5 code	Sample name	Code	Group	Geographical origin
78	46	Eclats d'Ambre	HM.EdA.SA	Moschata	GDA Sidi Amor
79	47	Felicia	HM.F.SA	Moschata	GDA Sidi Amor
80	48	Sidi Amor	HM.SA.SA	Moschata	GDA Sidi Amor
81	_	Pierre de Ronsard	PdR.SA	Climber	GDA Sidi Amor
82	_	Patrick de Carolis	PaCa.SA	Climber	GDA Sidi Amor
83	_	Jasmina	J.SA	Climber	GDA Sidi Amor
84	_	Red Parfum	RP.SA	Climber	GDA Sidi Amor
85	_	Parure d'or	PO.SA	Climber	GDA Sidi Amor
86	_	Baby Remantica	BR.SA	Climber	GDA Sidi Amor
87	_	Peace and love	PAL.SA	Climber	GDA Sidi Amor
88	58	Madame Isaac Pereire	RB.MIP.SA	Climber	GDA Sidi Amor
89	_	Madame Alfred Carriere	MAC.SA	Climber	GDA Sidi Amor
90	_	Madame Solvay	MSo.SA	Climber	GDA Sidi Amor
91	_	Alhambra	L.SA	Climber	GDA Sidi Amor
92	_	Indica Major	IM.SA	Climber	GDA Sidi Amor
93	_	Pink Cloud	PC.SA	Climber	GDA Sidi Amor
94	_	Annapurna	A.SA	hybrid Tea	GDA Sidi Amor
95	_	Roxane	R.SA	hybrid Tea	GDA Sidi Amor
96	_	Modern Rose	MR.BNG	Shurb.	NGBT
97	_	Marianne James	MJ.SA	Shurb.	GDA Sidi Amor
98	_	Landlust	La.SA	Shurb.	GDA Sidi Amor
99	_	Modern Rose	MR.B	Shurb.	Beja
100	_	Sourire Orchidie	SO.SA	Shurb.	GDA Sidi Amor
101	_	Mathilde Seigner	MSe.SA	Shurb.	GDA Sidi Amor
102	_	Madame Juliette Guillot	HF.MJG.SA	Floribunda.	GDA Sidi Amor
103	_	Centenaire de Lourdes	HF.CdL.SA	Floribunda.	GDA Sidi Amor
104	_	Domaine de Courson	HF.DdC.SA	Floribunda.	GDA Sidi Amor
105	_	Westerland	HF.W.SA	Floribunda.	GDA Sidi Amor
106	49	*Rosa sempervirens*	RS.T(2)	Wild	Tabbeba
107	50	*Rosa sempervirens*	RS.S	Wild	Sejnane
108	51	*Rosa Banksiae*	HC.B.SA	Wild	GDA Sidi Amor
109	52	*Rosa sempervirens*	RS.T(1)	Wild	Tabbeba
110	53	*Rosa sempervirens*	RS.N	Wild	Nefza
111	54	*Rosa moschhata*	RM.SA	Wild	GDA Sidi Amor
112	55	*Rosa canina*	RC.B(1)	Wild	Djeba
113	56	*Rosa canina*	RC.B(2)	Wild	Djeba
114	57	*Rosa canina*	RC.S	Wild	Sfax

Abbreviations list:

GDA Sidi Amor: Agricultural Development Group of Sidi Amor

NGBT: National Gene Bank of Tunisia
