# Supplementary figures and images for: Genetic Diversity and Relationships Among Tunisian Wild and Cultivated Rosa L. Species
Source: Plants (Basel). 2024 Dec 20;13(24):3563. doi: 10.3390/plants13243563 (PMC11678506; doi:10.3390/plants13243563)

**Figure S1.** Electrophoretic Profile of SSR Marker (RhB303) PCR Products on Metaphor Agarose Gel

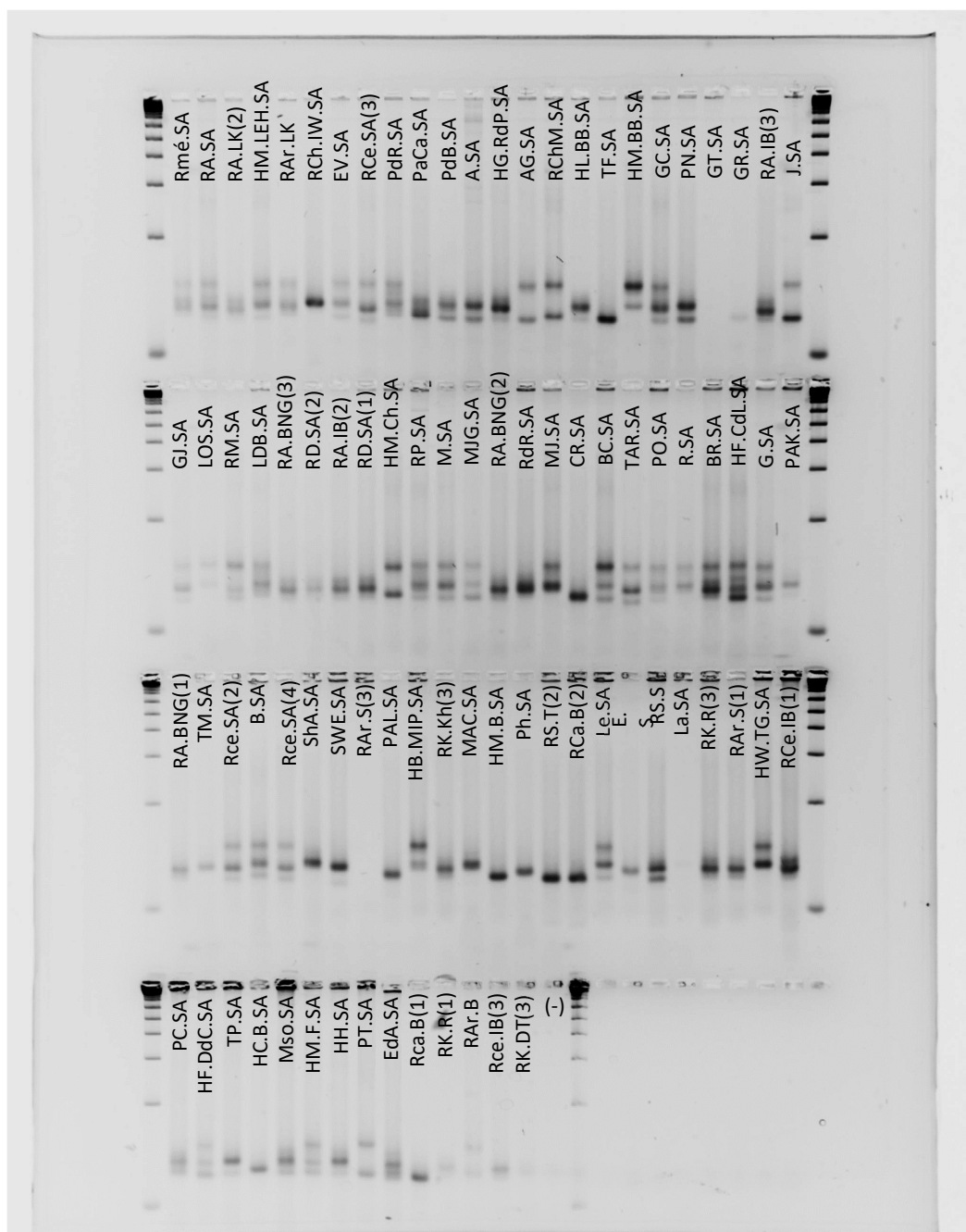

Supplement: Supplementary file 1 [file plants-13-03563-s001.zip › Figure S1.pdf]
